# Targeting the ROS/PI3K/AKT/HIF‐1α/HK2 axis of breast cancer cells: Combined administration of Polydatin and 2‐Deoxy‐d‐glucose

**DOI:** 10.1111/jcmm.14276

**Published:** 2019-03-28

**Authors:** Tao Zhang, Xinying Zhu, Haichong Wu, Kangfeng Jiang, Gan Zhao, Aftab Shaukat, Ganzhen Deng, Changwei Qiu

**Affiliations:** ^1^ Department of Clinical Veterinary Medicine College of Veterinary Medicine, Huazhong Agricultural University Wuhan People's Republic of China

**Keywords:** 2‐deoxy‐D‐glucose, breast cancer, glycolysis, PI3K/AKT, Polydatin

## Abstract

It is well established that cancer cells depend upon aerobic glycolysis to provide the energy they need to survive and proliferate. However, anti‐glycolytic agents have yielded few positive results in human patients, in part due to dose‐limiting side effects. Here, we discovered the unexpected anti‐cancer efficacy of Polydatin (PD) combined with 2‐deoxy‐D‐glucose (2‐DG), which is a compound that inhibits glycolysis. We demonstrated in two breast cell lines (MCF‐7 and 4T1) that combination treatment with PD and 2‐DG induced cell apoptosis and inhibited cell proliferation, migration and invasion. Furthermore, we determined the mechanism of PD in synergy with 2‐DG, which decreased the intracellular reactive oxygen (ROS) levels and suppressed the PI3K/AKT pathway. In addition, the combined treatment inhibited the glycolytic phenotype through reducing the expression of HK2. HK2 deletion in breast cancer cells thus improved the anti‐cancer activity of 2‐DG. The combination treatment also resulted in significant tumour regression in the absence of significant morphologic changes in the heart, liver or kidney in vivo. In summary, our study demonstrates that PD synergised with 2‐DG to enhance its anti‐cancer efficacy by inhibiting the ROS/PI3K/AKT/HIF‐1α/HK2 signalling axis, providing a potential anti‐cancer strategy.

## 1 INTRODUCTION

While recent advances have been made in cancer therapies and cancer diagnostic methods, most cancer remains an incurable disease with highly mortality in many parts of the world.^1^, ^2^ Previous studies suggest that patient death results mostly from cancer cell metastasis to distant organs and chemotherapy plays an imperative role in metastatic cancer treatment.^3^, ^4^ Several agents that are currently used for chemotherapy, such as vinorelbine, Paclitaxel and anthracyclines, obviously induce cancer cell apoptosis, but they also cause excessive damage to normal cells, leading to various serious side effects.^5^, ^6^ Thus, the development of novel therapeutic strategies that are safe and effective depends on a deeper understanding of the differences between cancer cells and normal cells. One of the fundamental biochemical characteristics in most malignant cancer cells is that they produce energy by an increased rate of aerobic glycolysis, instead of oxidative phosphorylation.^7^ Therefore, alteration of the manner of energy metabolism, a biochemical fingerprint of cancer cells, has been recognised as one of the ‘‘hallmarks of cancer’’. A series of cancer cell behaviours, such as infinite proliferation and metastasis, consume a large amount of energy. To acclimatise to this situation, cancer cells must elevate their glucose up‐take due to glycolysis being less effective than oxidative phosphorylation in the adenosine triphosphate (ATP) yield.^8^ Thus, targeting glycolysis is a promising therapeutic strategy for metastatic cancer. Emerging research suggests that an inhibition of glycolysis kills the malignant cells with a mild effect or even no effect on normal cells.^9^, ^10^ In fact, various types of drugs that inhibit glycolysis have been widely used in the treatment of malignant cancer.

2‐deoxy‐D‐glucose (2‐DG) is one of the most effective anti‐glycolytic agents. It is phosphorylated by hexokinase (HK), which is the first rate‐limiting enzyme of glycolysis and subsequently inhibits the pentose‐phosphate pathway (NAPDH) and ATP generation.^9^, ^11^ 2‐DG can change the redox state of the cell, or the generation of free radicals and then disorder the cell cycle and induce apoptosis.^12^ Previous studies have shown that 2‐DG is an effective anti‐cancer agent in cellular systems and in animal models.^13^, ^14^ However, 2‐DG monotherapy has yielded few positive results in mouse xenografts and human patients, most likely due to dose‐limiting side effects or the activation pro‐survival pathways in cancer cells.^15^, ^16^

**Figure 1:**
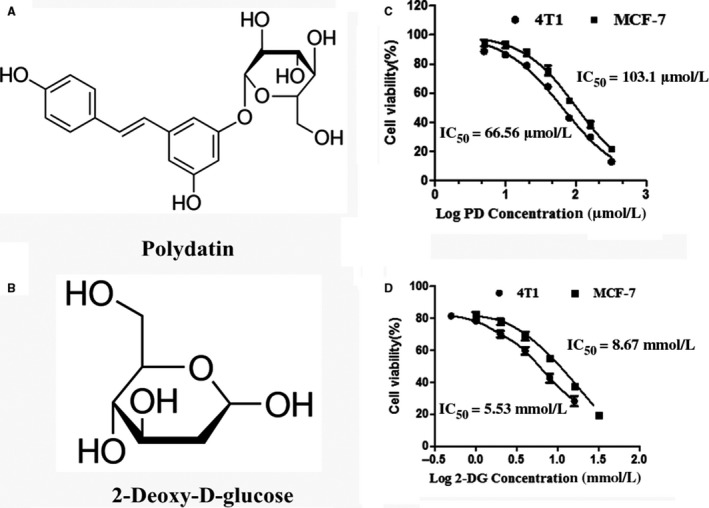
Polydatin (PD) and 2‐deoxy‐D‐glucose (2‐DG) inhibit breast cancer cell viability. (A and B) The chemical structures of polydatin and 2‐Deoxy‐D‐glucose. (C and D) The 24 h IC_50_ values of PD and 2‐DG were calculated in 4T1 and MCF‐7 cells. Cell viability was determined via cell counting Kit‐8. The experiments were performed three times

Recent studies in cancer chemotherapy are focused on a combination of two or more drugs. Polydatin (PD, 3,4'‐5‐trihydroxystilbene‐3‐β‐D‐glucopyranoside, shown in Figure 1A), is extracted from the roots of *Polygonum cuspidatum *Sieb and can be detected in grape, peanut, hop cones, red wines, hop pellets, cocoa‐containing products, chocolate products and many daily diets, is widely applied in traditional Chinese therapy.^17^ Previous studies demonstrated that PD has antioxidant, anti‐inflammatory and anti‐cancer activities and it is mainly used in cardiovascular, inflammatory, neurodegenerative, metabolic and age‐related diseases.^18^, ^19^ PD exerts its anti‐cancer effect in a variety of ways, such as the regulation of reactive oxygen species (ROS)^20^ and inhibition of the PI3K/AKT pathway.^21^ Interestingly, some studies indicated that PI3K/AKT inhibitors enhanced the therapeutic efficacy of 2‐DG.^22^, ^23^ In addition, PD has received considerable attention for its beneficial effects on glucose and lipid regulation.^24^ Thus, whether PD can promote the anti‐cancer effects of 2‐DG by regulating glucose metabolism, inhibiting the PI3K/AKT pathway or other mechanisms has aroused great interest.

In the present study, we evaluated the anti‐cancer effect of PD, 2‐DG and their co‐treatment in breast cancer cell lines (4T1 and MCF‐7) and elucidated the underlying molecular mechanisms.

## 2 MATERIALS AND METHODS

### 2.1 Cell culture

The 4T1 Cells used in this study were obtained from the Chinese Academy of Sciences cell Bank (Shanghai, China) and MCF‐7 cells were purchased from the American Tissue Culture Collection (ATCC, Rockville, MD). All cell lines were grown in RPMI 1640 medium with 10% fetal bovine serum (FBS) and 1% penicillin–streptomycin at 37°C with 5% CO2. Cells were cultured in T25 cell culture flasks. When the cell density reached more than 80%, the cells were subcultured into 96‐well plates or 6‐well plates for the corresponding experiments.

### 2.2 Antibodies and reagents

Western blotting, immunofluorescence and immunohistochemistry were performed using the following antibodies: PI3K, phospho‐PI3K, AKT, phospho‐AKT, HK2, HIF‐α, β‐actin (Cell Signaling Technologies; Danvers, MA);cleaved‐PARP, Bax, Bcl‐2, MMP‐2, MMP‐9, VEGF, LDH‐A, Glut1 (Abcam, Cambridge, UK). 2‐deoxy‐D‐glucose (2‐DG, purity ≥ 99%) and polydatin (PD, purity > 99%) were obtained from Sigma‐Aldrich Chemical (Agent of Shanghai, China). The Apoptosis Assay Kit and FITC Annexin V Apoptosis Detection Kit with PI (propidiumiodide), N‐acetyl‐cysteine (NAC) and the PI3K/AKT inhibitor LY294002 was purchased from the Beyotime Institute of Biotechnology (Shanghai, China). The cell counting Kit‐8 was purchased (CCK‐8, Dojindo Laboratories, Minato‐ku, Tokyo, Japan). All of the other chemicals and reagents were of the highest commercial grade available.

### 2.3 Cell viability and proliferation assay

CCK‐8 was used to evaluate the viability and proliferation of breast cancer cell lines (4T1 and MCF‐7). Cells were seeded in 96‐well plates at a density of 5×10^4^ cells/ml. After treatment, cells were continuously cultured with 10 μL of CCK8 in each well at 37°C for 2 hours. Cell proliferation was measured through absorbance (optical density) with a microplate reader (Bio‐Rad Instruments, Hercules, CA) at 450 nm.

### 2.4 Cell migration assay

A Wound Healing Assay was used to assess cell migration. As a previous study described the method,^25^ the 4T1 and MCF‐7 cells were seed onto 6‐well plates. After treatment, wounding was performed by dragging a 200 μL pipette tip through the monolayer and then washing the cells twice. The 4T1 and MCF‐7 cells culture with serum‐reduced Opti‐MEM I medium (Invitrogen, CA), and wound closure images were photographed by an inverted microscope.

### 2.5 Flow cytometry apoptosis analysis

After treatment, cells were trypsinized and washed twice in cold phosphate‐buffered saline. Cells were stained using the Annexin‐V‐PE apoptosis detection kit (BD, San Diego, CA) according to the manufacturer's protocol and were analysed by FACS (BD).

### 2.6 Measurement of ROS production

The intracellular ROS level was measured by flow cytometry. Briefly, cells were seeded at a density of 1 × 10^6^ cells ml^−1^ into 6‐well plates and, after treatment as indicated, the intracellular ROS level was measured using the oxidative conversion of cell permeable 2′,7′‐dichlorofluorescein diacetate (DCFH‐DA, Beyotime, China) to fluorescent dichlorofluorescein (DCF). The cells were incubated with DCFH‐DA for 30 minutes at 37°C, then washed again and flow cytometry was performed.

### 2.7 Transient transfection

HK2 expression in 4T1 and MCF‐7 cells was ablated with siRNAs. A scrambled siRNA (siRNA‐NC) was used as a control (GenePharma, Shanghai, China). The siRNAs were transfected into cells using Lipofectamine 2000 (Invitrogen). The transduction efficiency was measured by real‐time PCR and western blotting.

### 2.8 RNA extraction and qPCR

Total RNA was extracted from collected cells using the TRIzol reagent (Invitrogen, USA) and reverse transcription to synthesise cDNA was performed using a reverse transcription kit (Takara, Japan). The primer sequences used are shown in Table 1. Quantitative real‐time PCR mixture reagents contains 1 μL forward and reverse primers, 1 μL of cDNA, 10 μL SYBR qPCR Mix (Roche, Swiss) and 7 μL TE water and on a StepOne Plus™ Real‐Time PCR System (BIO‐RAD, USA). The target genes expression level was evaluated using the 2^−ΔΔCt^ comparative approach.

**Table 1:** Primer sequence for qPCR

Gene	Primer sequence (5'‐3')	Product size (bp)
mus‐GAPDH	Forward: AACAGCAACTCCCACTCTTC	111
	Reverse: CCTGTTGCTGTAGCCGTATT	
mus‐Bax	Forward: GGAGATGAACTGGACAGCAATA	117
	Reverse: GAAGTTGCCATCAGCAAACAT	
mus‐Bcl‐2	Forward: GTGGATGACTGAGTACCTGAAC	125
	Reverse: GAGACAGCCAGGAGAAATCAA	
mus‐cIAP	Forward: AGGAGTCTTCCCACAGATGA	99
	Reverse: CTCTCGGTCCATACACACTTTAC	
mus‐XIAP	Forward: CCAGAATCCTATGGTGCAAGAA	123
	Reverse: GCAATCAGGACCTCAAGTGATAG	
Hum‐GAPDH	Forward: GGAGCGAGATCCCTCCAAAAT	111
	Reverse: GGCTGTTGTCATACTTCTCATGG	
Hum‐Bax	Forward: GTCACTGAAGCGACTGATGT	128
	Reverse: CTTCTTCCAGATGGTGAGTGAG	
Hum‐Bcl‐2	Forward: GTGGATGACTGAGTACCTGAAC	125
	Reverse: GAGACAGCCAGGAGAAATCAA	
Hum‐cIAP	Forward: GTGGCTGCATTTGGTGTTATC	115
	Reverse: GATCTTCAGACTCCTTGCTCTTC	
Hum‐XIAP	Forward: GAGGAACCCTGCCATGTATAG	111
	Reverse: GTGTAGTAGAGTCCAGCACTTG	

### 2.9 Western blot analysis

The total protein of cells or tissues were extracted with a RIPA lysis solution involving phosphatase inhibitors (Vazyme, Nanjing, China) and then separated by SDS‐PAGE and transferred to PVDF membrane. The membrane was incubated in blocking buffer (5% skim milk) and subsequently treated with primary antibody and secondary antibody. The protein expression levels were determined using the ECLPlus western blot Detection System.

### 2.10 *In vivo* experiment

Tumour formation in 8‐week‐old BALB/c female mice was used as a model. The mice were purchased at the Hubei Provincial Center for Experimental Animal Research (Wuhan, China). The animal protocol was approved by the Institutional Ethical Committee for Animal Care and Use of Huazhong Agricultural University. In line with the United States National Institutes of Health published experimental animal care and use.

Approximately 2.0 × 10^7^ 4T1 cells without any contamination were harvested and suspended in 100 μL of PBS, then subcutaneously injected into the mouse's fourth breast pad. Two weeks after the injection, mice were randomised into four groups and treated as follows: PD (100 mg/kg ip every other day for 3 weeks), 2‐DG (100 mg/kg ip every other day for 3 weeks), a combination of both, or saline as an untreated vehicle. The size of subcutaneous tumours and weight of the mice were recorded every 2 days. Tumour volume (V) was calculated according to the formula V = 0.5 × L × W^2^, where L is the greatest diameter and W is the diameter at the point perpendicular to L. At the end of treatment, mice were sacrificed and the tumour tissues and major vital organs from each group were harvested and immersed in 4% paraformaldehyde. The tumours were removed and used for immunohistochemical staining.

### 2.11 Histopathological assessment and immunohistochemistry

Tumour tissues and the major vital organs, such as the heart, liver and kidneys, of mice were isolated and fixed in 10% formalin. The tissues were dehydrated, paraffin embedded and then cut into 5‐μm‐thick sections for hematoxylin and eosin (H&E) staining.

Immunohistochemistry detection using the following primary antibodies: anti‐VEGF, anti‐Ki67, anti‐HIF‐α and anti‐HK2 was performed on paraffin sections. The staining processes were performed according to standard methods. The sections were observed using an optical microscope (Olympus, Japan).

### 2.12 Immunofluorescence assay

The breast cancer cells were inoculated into a 6‐well‐plate. After treatment, the cells were fixed with 4% (v/v) paraformaldehyde for 20 minutes, permeabilised with 0.2% Triton X‐100 for 10 minutes and then blocked with 5% BSA for 1 hour, followed by incubated with the primary antibodies at 4°C overnight. After three washes in PBS, the cells were incubated with the FITC‐labelled goat anti‐rabbit IgG antibody for 1 hour. Nuclei were stained with 4,6‐diamidino‐2‐phenylindole (DAPI, Beyotime, China) for 10 minutes, and observed using fluorescence microscopy (Olympus, Japan).

### 2.13 TUNEL assay

The TdT‐UTP nick end labelling (TUNEL) assay was performed using a TUNEL assay kit (Roche Diagnostics GmbH, Germany) according to the manufacturer's instructions. Briefly, the tumour tissues underwent routine deparaffinization and dehydration. They were then digested with 20 μg/mL proteinase K for 15 minutes, rinsed with PBS and incubated with TUNEL reagents containing terminal deoxynucleotidyl transferase (TdT) and fluorescent isothiocyanate dUTP for 2 hours at 37°C. Finally, the samples were stained with DAPI for 30 minutes to evaluate the cell nucleus. The apoptotic cells were recognised with dual TUNEL and DAPI staining under a fluorescence and UV light microscope.

### 2.14 Statistical analysis

All values are presented as the means ± SEM for three independent experiments. The intergroup differences were determined by a two‐way ANOVA and Student's *t* test. A value of *P* ≤ 0.05 was considered statistically significant and *P* ≤ 0.01 was highly statistically significant. All results were analysed using GraphPad Prism 5.

## 3 RESULTS

### 3.1 PD and 2‐DG demonstrates cytotoxicity on breast cancer cells

As previously reported, PD and 2‐DG are cytotoxic to a variety of cancer cells.^12^, ^21^ In this study, testing of the cytotoxicity of PD and 2‐DG on breast cancer cells 4T1 and MCF‐7 was conducted using the CCK‐8 kit (Figure 1B,C). The results showed that cell viability decreased in a dose‐dependent manner. The potency of PD was similar in both cell lines (4T1, IC_50_: 66.56 μmol/L and MCF‐7, IC_50_: 103.1 μmol/L). The IC_50_ for 2‐DG in 4T1 and MCF‐7 was about equal at 5.53 mmol/L and 8.67 mmol/L, respectively. The difference in IC_50_ values for PD in both cell lines was not significant. Based on these findings, 100 μmol/L PD and 5 mmol/L (4T1) or 10 mmol/L (MCF‐7) 2‐DG were used for further studies, as this is the approximate IC_50_ value for both cell lines.

### 3.2 PD combined with 2‐DG displays a potent anti‐cancer activity in vitro

**Figure 2:**
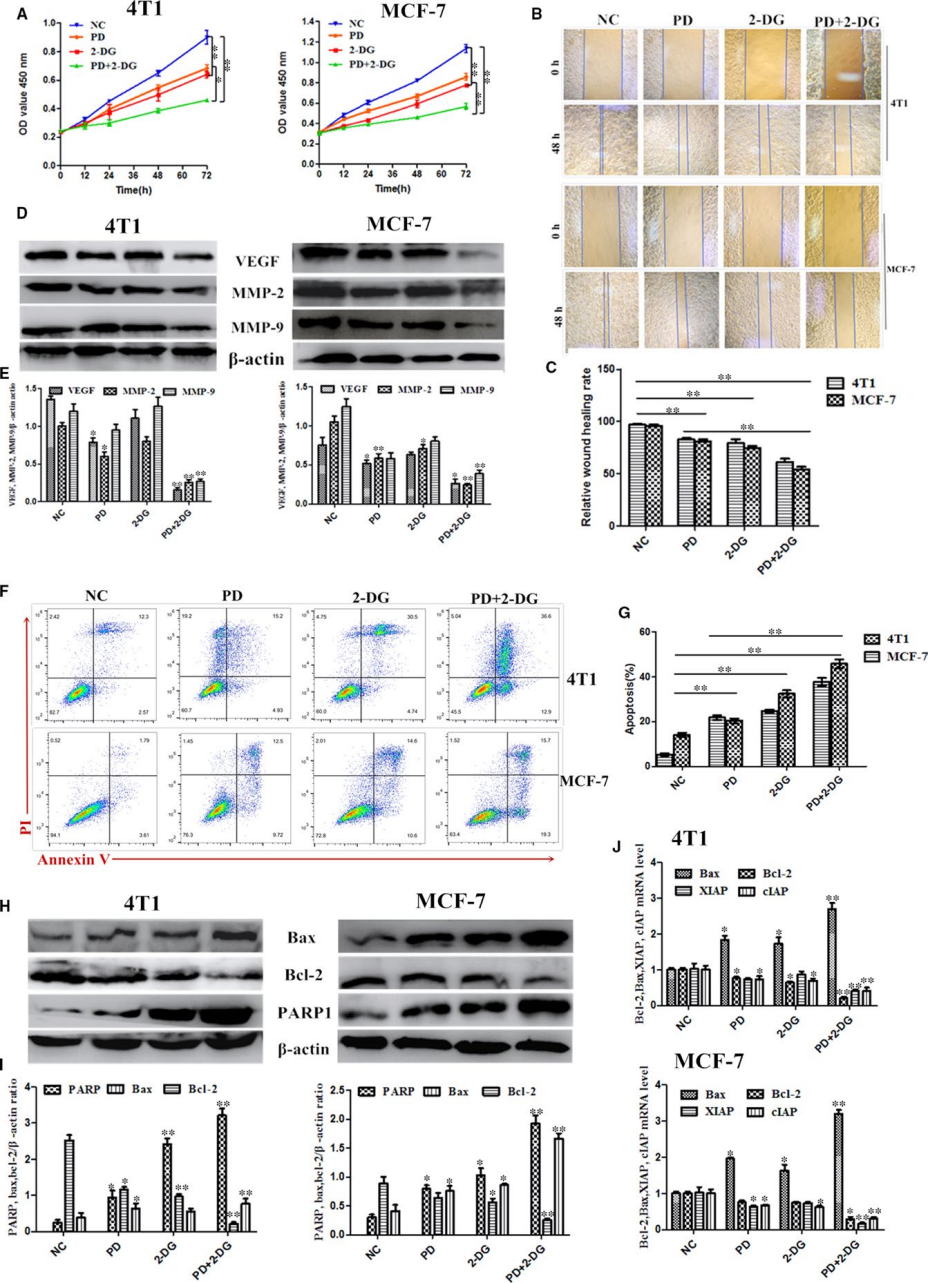
Polydatin (PD) synergizes with 2‐deoxy‐D‐glucose (2‐DG) to decrease cell proliferation in 4T1 and MCF‐7 cells. (A) Cell counting Kit‐8 kits were used to assess the proliferation of 4T1 and MCF‐7 cells treated with PD, 2‐DG or PD combined with 2‐DG at 0 h, 12 h, 24 h, 48 h and 72 h. (B and C) Wound healing assays of 4T1 and MCF‐7 cells after PD and 2‐DG treatment alone or in combination. Representative images depicting the beginning (t = 0 h) and the end (t = 48 h) of the recording period are shown. (D and E) The protein expression levels of VEGF, MMP‐2 and MMP‐9 were detected using western blots. β‐actin was used as an internal control. (F and G) Cell apoptosis assays of 4T1 and MCF‐7 cells treated with PD, 2‐DG or their combination using FACS. Cells were collected and labelled with Annexin V‐FITC and PI. (H and I) Western blot analyses of Bax, Bcl‐2 and PARP1 from cells treated with PD, 2‐DG or their combination for 24 h. β‐actin was used as an internal control. (J) The mRNA expression levels of Bax, Bcl‐2, XIAP and cIAP were detected in 4T1 and MCF‐7 cells by RT‐PCR. GAPDH was used as an internal control. All results are expressed as the mean ± SEM of three independent experiments. The symbols * and ** denote significant differences of *P* < 0.05 and *P* < 0.01, respectively

PD is known to inhibit proliferation and induce apoptosis in breast cancer and colorectal cancer.^26^, ^27^ Therefore, we investigated whether a combined treatment with 2‐DG might affect the migration, proliferation and apoptosis of 4T1 and MCF‐7 cells. Compared to untreated controls, PD and 2‐DG significantly inhibited cell proliferation and migration, but their individual inhibitory capacities were both lower than that of the PD and 2‐DG combination group (Figure 2A‐C). Previous studies have indicated that matrix metalloproteinase‐2 (MMP‐2) and matrix metalloproteinase‐9 (MMP‐9) disrupt the extracellular matrix (ECM) and then induce cancer cell migration and invasion.^28^, ^29^ In vitro, increased autocrine vascular endothelial growth factor (VEGF) is also considered to be one of the hallmarks of cancer invasion.^30^ Our results indicated that the combined treatment of 2‐DG and PD led to a significant inhibition of MMP9, MMP2 and VEGF expression compared with the control or individual treatment groups (Figure 2D,E).

Apoptosis is a crucial process in the maintenance and progression of cancer cells. Therefore, we monitored the effects of PD and 2‐DG induced breast cancer cell apoptosis by FACS analysis. The results indicated that the apoptotic rate of breast cancer cells was markedly lowered in the PD and 2‐DG co‐treatment group compared to vehicle‐treated and individual treatment groups (Figure 2F,G). In addition, we also evaluated the protein levels of PARP1, Bax and Bcl‐2, which are typical apoptotic proteins in cancer cells (Figure 2H,I). The mRNA levels of Bax, Bcl‐2, cIAP and XIAP were also detected by RT‐PCR (Figure 2J). These results were consistent with the above conclusions. In summary, synergistic actions between the PD and 2‐DG components contribute to an anti‐tumour property in vitro.

### 3.3 The PD and 2‐DG combination treatment inhibits the PI3K/AKT prosurvival signalling pathway

**Figure 3:**
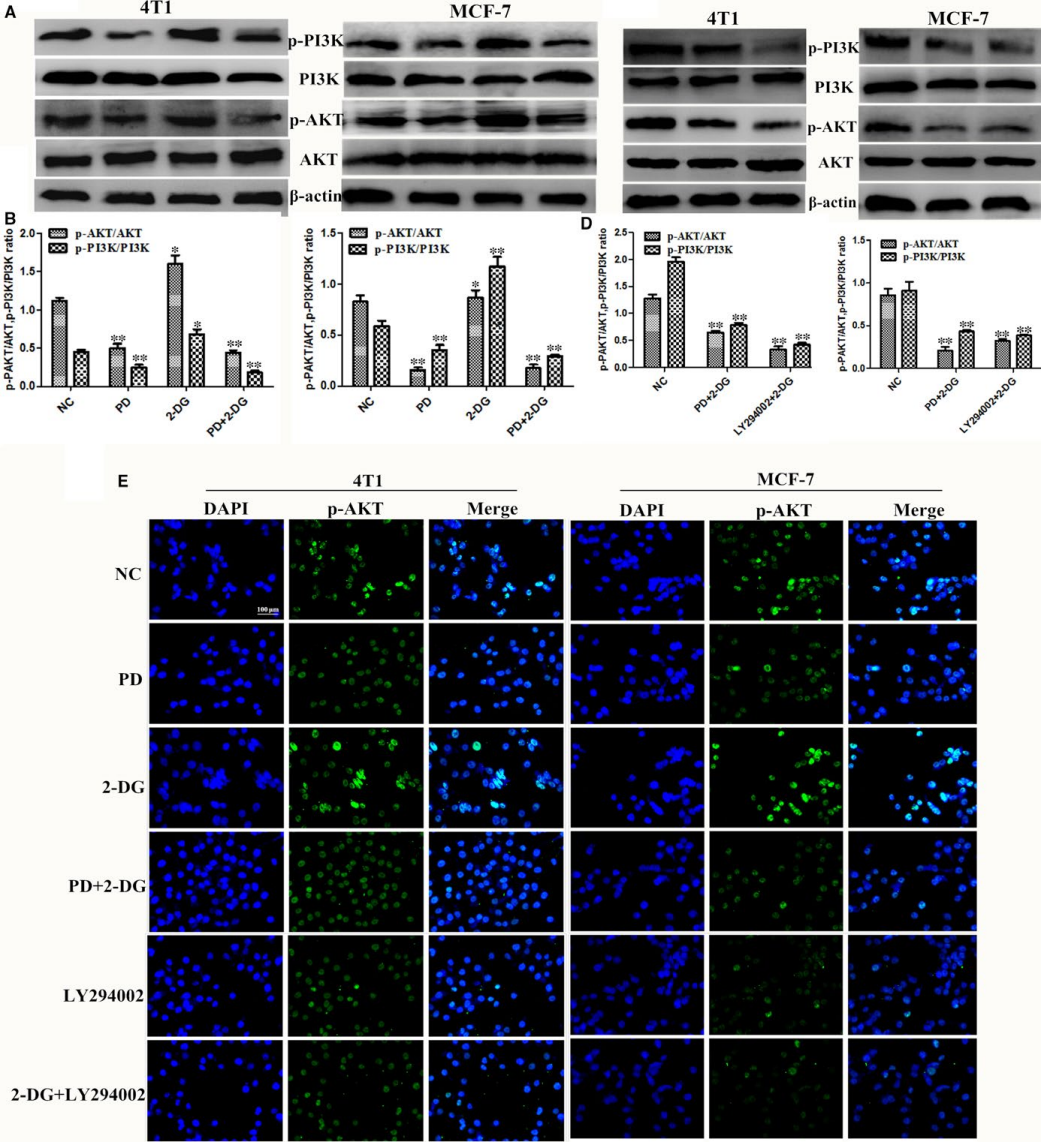
Polydatin (PD) combined with 2‐deoxy‐D‐glucose (2‐DG) inhibits the PI3K/AKT signalling pathway. The 4T1 and MCF‐7 cells were treated for 24 h with either 2‐DG alone or PD or LY294002 (2 μmol/L) or with a combination of both agents alone. (A‐D) The protein expression levels of p‐PI3K, PI3K, p‐AKT and AKT were detected by western blot. β‐actin was used as an internal control. (E) Immunofluorescence staining of p‐AKT in 4T1 and MCF‐7 cells treated with PD, 2‐DG and their combination. Scale bar: 100 μm (n = 3). All results are expressed as the mean ± SEM of three independent experiments. The symbols *and **denote significant differences of *P* < 0.05 and *P* < 0.01, respectively

To further understand the molecular mechanisms by which PD and 2‐DG exert their synergistic effects on breast cancer cell lines, we next explored whether other major survival‐promoting signalling pathways may be involved. Previous studies have shown that 2‐DG induced prosurvival signalling through AKT activation and then reduced cytotoxicity.^22^, ^31^ Therefore, we examined the expression of PI3K/AKT critical signalling proteins in 2‐DG and PD combination treated 4T1 and MCF‐7 cells. The rates of p‐PI3K/PI3K and p‐AKT/AKT were dramatically decreased in the cotreatment group compared the control group or the individual agent treatment groups (Figure 3A,B). In addition, we also found that 2‐DG co‐treatment with LY294002 (Standard PI3K/AKT Inhibitor) achieved these results (Figure 3C,D). This indicates that the inhibition of the PI3K/AKT signalling pathway by PD curbs the 2‐DG‐induced prosurvival signalling. We further confirmed this result by immunofluorescence (Figure 3E).

### 3.4 PD inhibits ROS generation, a critical regulatory factor of the PI3K/ AKT pathway

**Figure 4:**
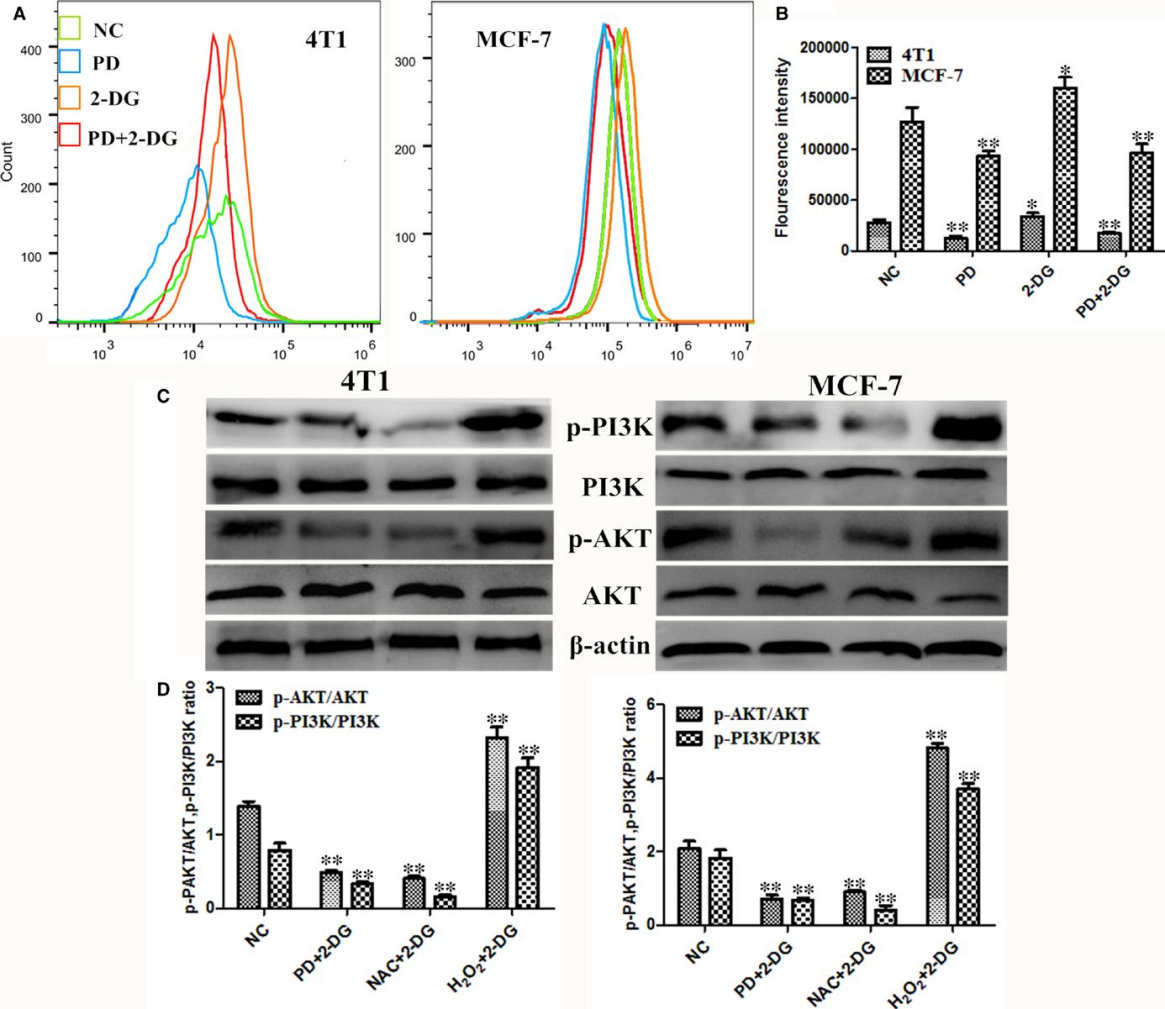
Polydatin (PD) inhibits the PI3K/AKT signalling pathway although the reduction of intracellular ROS levels. (A and B) After treatment, the intracellular ROS levels of breast cancer cells were measured by staining with DCFH‐DA (10 mmol/L) for 30 min, followed by flow cytometry. (C) The 4T1 and MCF‐7 cells were treated with 10 mmol/L NAC for 2 h prior to 2‐DG treatment in the NAC + 2‐DG group and 10 μmol/L H_2_O_2_ combined with 2‐DG in the H_2_O_2_ + 2‐DG group for 24 h. The protein expression levels of p‐PI3K, PI3K, p‐AKT and AKT were detected by western blot. β‐actin was used as an internal control. The symbols * and ** denote significant differences of *P* < 0.05 and *P* < 0.01, respectively

Further studies were performed to determine the upstream regulators of the PI3K/AKT pathway and its downstream targets. As mentioned before, previous studies revealed that PD possesses antioxidant pharmacological effects.^17^, ^32^, ^33^ Therefore, we hypothesised that reactive oxygen species (ROS) are a critical upstream mediator of the PI3K/AKT pathway under PD and 2‐DG co‐treatment. As expected, PD decreased the intracellular ROS level and attenuated 2‐DG‐induced ROS elevation in both cell lines (Figure 4A,B). As 2‐DG‐induced ROS production was abrogated by the ROS scavenger NAC, we then examined the activity of PI3K/AKT. We found that NAC inhibited PI3K/AKT compared to other groups in breast cancer cells (Figure 4C,D). Furthermore, H_2_O_2_ prevented the inhibition of PI3K/AKT by the PD and 2‐DG combination (Figure 4C,D), suggesting that PD reduces the 2‐DG‐induced pro‐survival PI3K/AKT signalling pathway by reducing intracellular ROS levels.

### 3.5 The synergistic anti‐cancer effect of PD and 2‐DG is through the suppression of HK2 in breast cancer cells

**Figure 5:**
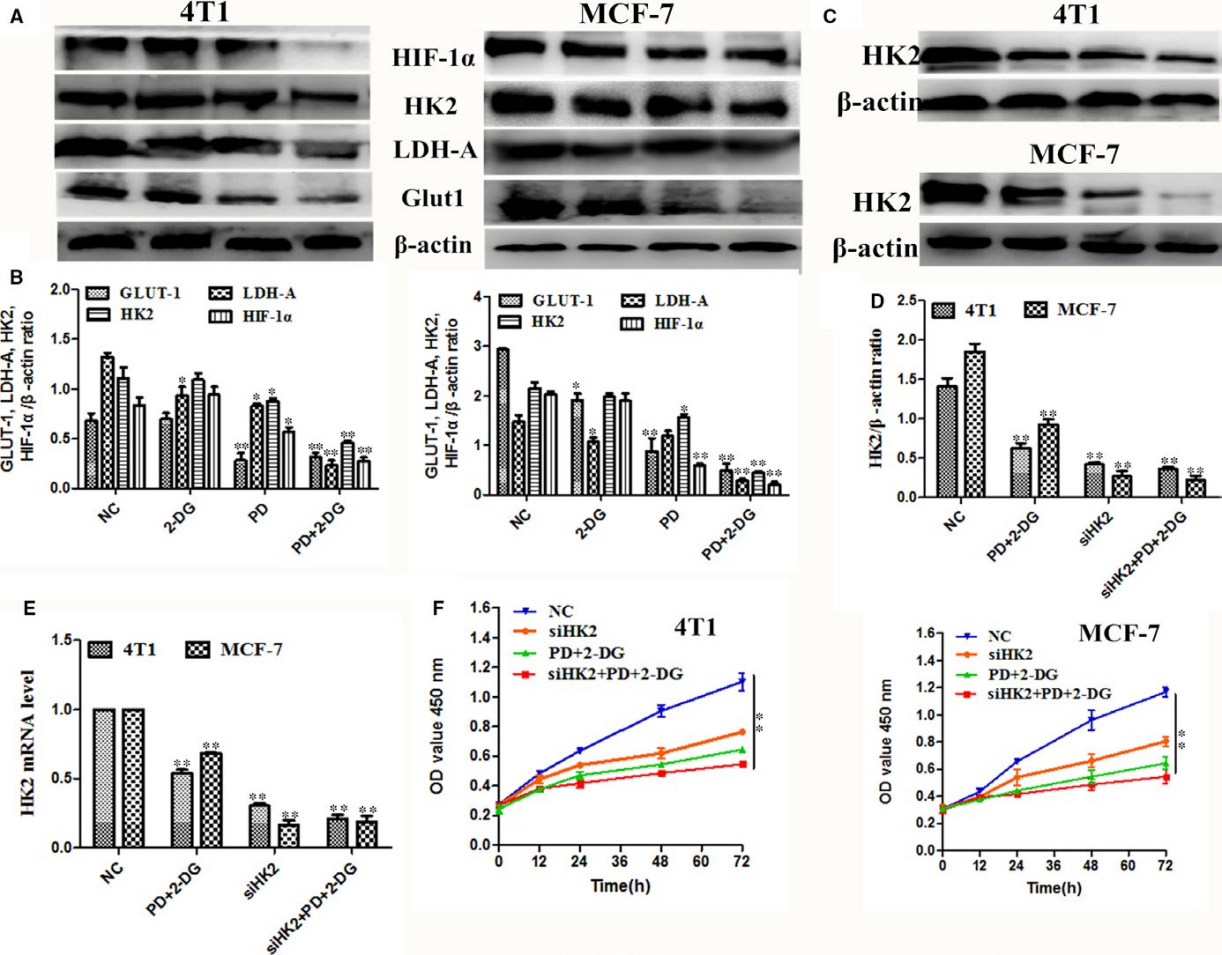
Combined treatment with Polydatin (PD) and 2‐deoxy‐D‐glucose (2‐DG) inhibits the glycolytic phenotype in breast cancer cells. (A and B) The protein expression levels of GLUT1, LDH‐A, HK2 and HIF‐1α were detected using western blots. β‐actin was used as an internal control. (C and E) The protein expression levels of HK2 in si‐HK2 and si‐NC transfected 4T1 and MCF‐7 cells were determined using Western blot. β‐actin was used as an internal control. (F) Cell counting Kit‐8 kits were used to assess the proliferation of 4T1 and MCF‐7 cells after transfected with siHK2 and combination treatment with PD and 2‐DG at 0 h, 12 h, 24 h, 48 h and 72 h. The symbols * and ** denote significant differences of *P* < 0.05 and *P* < 0.01, respectively

A typical hallmark of malignant cells is their capacity to perform glycolysis at a higher rate.^7^ The structural features of 2‐DG prompted us to evaluate the glycolytic phenotype in PD and 2‐DG co‐treated breast cancer cells. The results indicated that the first step rate limiting enzyme, glycolysis hexokinase (HK2), which is overexpressed in malignant cells,^34^ had significantly decreased protein levels following co‐treatment with PD and 2‐DG (Figure 5A,B). In further studies we examined other markers of glycolysis, such as glucose transporter 1 (GLUT1) and Lactate dehydrogenase A (LDH‐A) (Figure 5A,B), and the results were consistent with the changes of HK2. In addition, we noticed an aberrant overexpression of hypoxia‐inducible factor 1α (HIF1α) in many different cancers and HK‐2 is one of its downstream transcriptional targets.^35^, ^36^ In this study, we also found that a combination of PD and 2‐DG inhibited the protein expression of HIFIα in 4T1 and MCF‐7 cells (Figure 5A,B). In addition, we also found that targeting HK2 with siRNA can block breast cancer cell survival and this result was consistent with PD and 2‐DG co‐treatment (Figures 5C5, 6, 7). Overall, the PD and 2‐DG combination inhibits glycolytic metabolism by suppressing HIF1α/HK2 in breast cancer cells.

**Figure 6:**
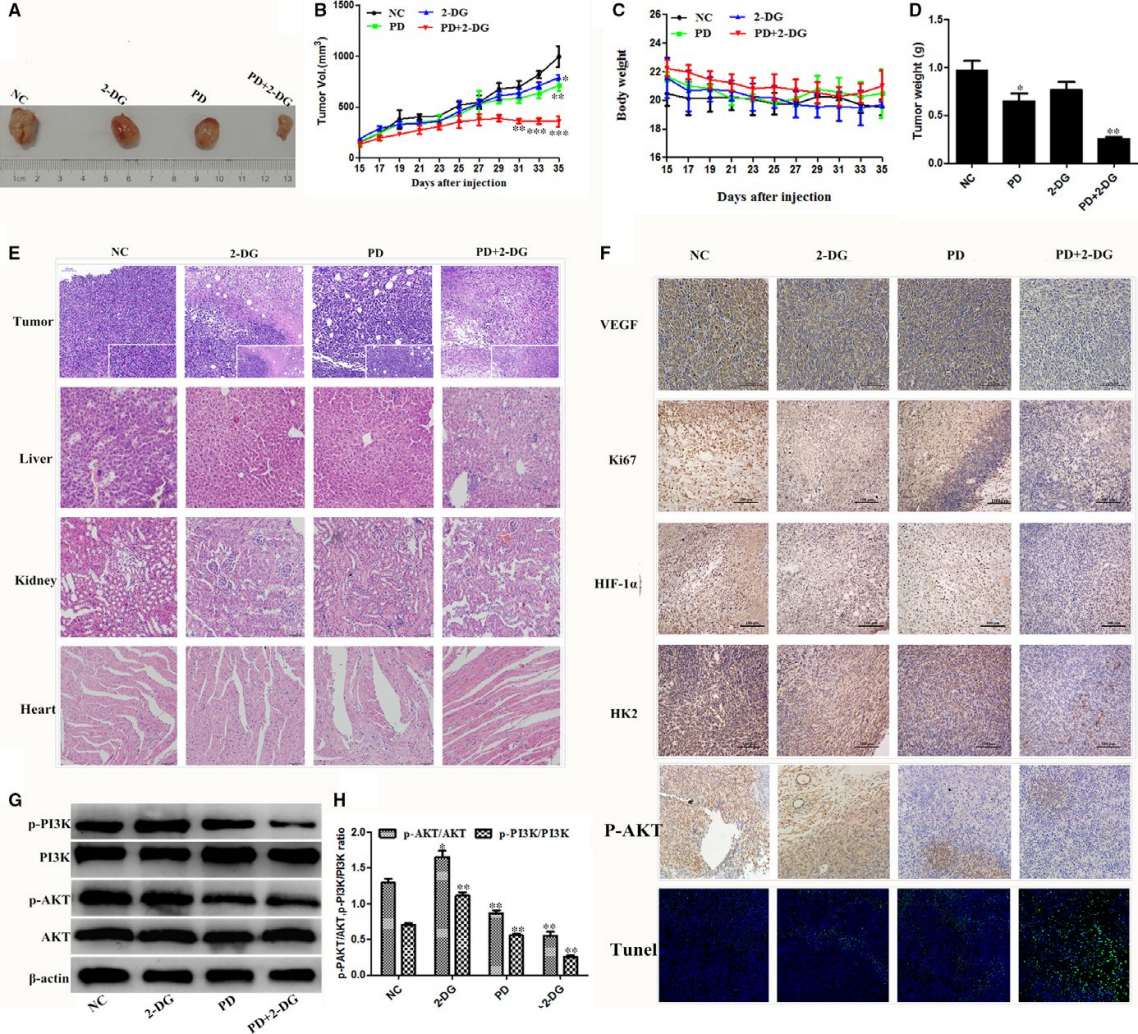
In vivo, Polydatin (PD) combined with 2‐DG inhibits xenograft tumour growth. The BALB/c female mice were divided into four groups and injection of the indicated drugs began on the fifteenth. Every two days, the tumour volume (A and B) and body weight (C) were measured (n = 5 per group). On day 35, the mice were sacrificed, samples were collected and the tumour weight was measured (D). Data shown as the mean ± SEM. The symbols *, ** and *** denote significant differences of *P* < 0.05, *P* < 0.01 and *P* < 0.001 versus the control group, respectively. (E) Representative panels of H&E staining of tumour and major vital organs, such as the heart, liver and kidneys, of mice (Scale bar 100 μm). (F) Representative immunohistochemical staining for VEGF, Ki67, HIF‐1α, HK2 and p‐AKT in tumours from the xenograft mouse model (Scale bar 100 μm). Apoptosis in the tumour sections was examined by TUNEL staining. Blue spots represent cell nuclei and green spots represent TUNEL‐positive cells (200 magnifications, Scale bar 50 μm). G. The phosphorylation levels of PI3K and AKT were determined by western blotting. β‐actin served as an internal control. Data represent the mean ± SEM of three independent experiments. The symbols * and ** denote significant differences of *P* < 0.05 and *P* < 0.01 versus the control group, respectively

**Figure 7:**
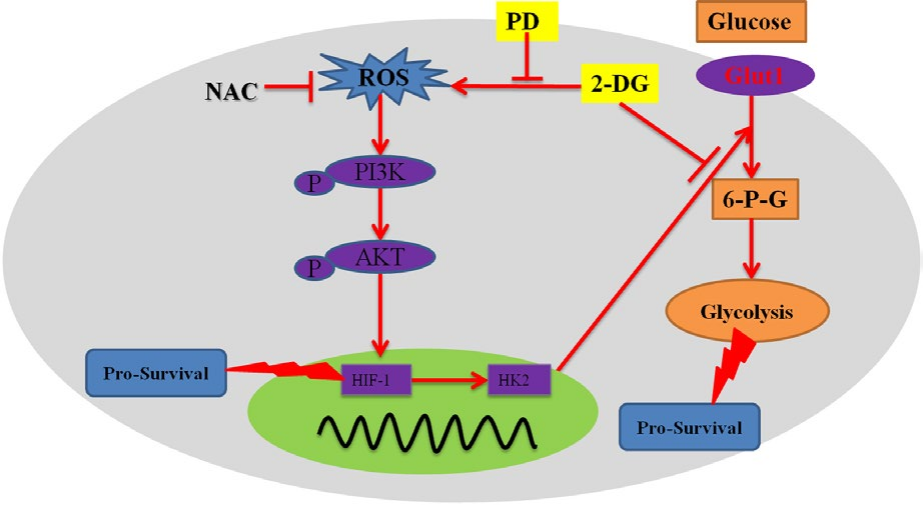
Schematic illustration delineating the role of Polydatin (PD)/2‐deoxy‐D‐glucose (2‐DG) combination therapy. 2‐DG alone can competitively inhibit the HK2‐induced conversion of glucose to 6‐P‐G. This leads to a decrease in the rate of glycolysis of the cells, an insufficient production of ATP and the induction of apoptosis. However, 2‐DG activates PI3K/AKT pro‐survival signalling. A reduction of intracellular ROS by PD blocks the 2‐DG induced PI3K/AKT activation. On one hand, the inhibition of PI3K/AKT down‐regulates the transcription of HIF‐1α and then inhibits the pro‐survival signal

### 3.6 The effects of PD and 2‐DG on tumour growth: murine syngeneic tumour models

To further verify the correctness of the in vitro conclusions, a murine syngeneic breast tumour model was used. The combined treatment of PD and 2‐DG led to a significant decrease in the tumour volume and weight compared with the control mice after 3 weeks of treatment (Figure 6A‐C). Furthermore, no evident histopathological abnormalities were observed in the vital organs, such as the heart, liver and kidneys, by HE staining (Figure 6E). We then evaluated cell proliferation (Ki67) and angiogenic activity (VEGF) by immunohistochemistry in the tumour sections. The combination treatment showed obviously anti‐proliferative and anti‐angiogenic activity (Figure 6F). TUNEL and apoptosis factor expression analyses further revealed that the PD and 2‐DG combination treatment effectively promoted apoptosis in homotransplant tumours (Figure 6F‐H). Moreover, in vivo*,* we validated the mechanism of the anti‐cancer effect of the PD and 2‐DG combination treatment and the results showed that the combination significantly inhibited the PI3K/AKT signal pathway (Figure 6I,J). Taken together, these observations indicate that the PD and 2‐DG combination treatment exerts a superior anti‐cancer activity both in vitro and in vivo.

## 4 DISCUSSION

The pivotal role of glycolysis in cancer onset and progression has been recently recognised.^37^, ^38^ Therefore, correcting the level of glycolysis of malignant cancer cells is one of the emerging research hotspots in cancer chemotherapy. However, the agents that specifically inhibit glycolytic metabolism have not yielded the expected effect in cancer patients, most likely due to cancer cells developing resistance to the agents or to dose‐limiting side effects.^11^, ^16^ Combinational chemotherapies have been widely used to minimise acquired resistance.^39^ In this study, we demonstrate that the synergy between PD and 2‐DG restrains tumour growth due to the inhibition of the pro‐survival ROS/PI3K/AKT/HIF1α/HK2 pathway (Figure 7). The effects of PD and 2‐DG combination treatment on cancer cell growth are striking in so far as the combination treatment directly abolished glycolysis, dramatically reduced metastasis and induced the apoptosis of MCF‐7 and 4T1 cells.

The glucose analogue, 2‐DG, which is phosphorylated by hexokinase, competes with 6‐phosphate glucose (6‐P‐G) to inhibit glycolysis, resulting in cancer cell death due to cell energy ATP deprivation.^9^, ^11^ Polydatin, also named piceid (PD), has many biomedical properties, such as anti‐platelet aggregation, antioxidative, anti‐cancer, anti‐inflammatory and immune‐regulating functions.^40^ In this study, we found that 2‐DG and PD both inhibited cell viability in 4T1 and MCF‐7 (Figure 1) and that PD combined with 2‐DG obviously induced apoptosis and led to a reduction in cell proliferation and migration in 4T1 and MCF‐7 compared to the individual treatment groups (Figure 2). However, previous studies have shown that 2‐DG induces the phosphorylation of AKT and the efficacy of clinical trials has previously been limited by the systemic toxicity.^41^, ^42^ The activation of AKT is commonly observed in a variety of cancers and seems to be intricately associated with aerobic glycolysis, proliferation and invasiveness.^43^, ^44^ In this study, we found that the mechanism of action of the treatment combination is that PD blocks the activation of the PI3K/AKT pathway by 2‐DG (Figure 3).

Under physiological conditions, various redox systems ensure that ROS are appropriately utilised to accomplish specific functions, such as signalling and protein regulation.^45^ It is generally accepted that ROS are tumour suppressors that are implicated in tumorigenesis, progression and survival phenotypes.^45^, ^46^ Considering the relevance of the role played by ROS in cancer, we evaluate the intracellular ROS level after PD and 2‐DG treatments in 4T1 and MCF‐7 cells. In this study we demonstrated that the PD and 2‐DG combination treatment decreased ROS production and then inhibited PI3K/AKT activation (Figure 4). Furthermore, H_2_O_2_ can rescue the effect of the co‐treatment on PI3K/AKT. All of the results indicated that ROS is a critical mediator of the combination's effects on PI3K/AKT pathway. It should be noted that a few studies have shown that AKT was also found to be down‐regulated by ROS in cancer cells.^47^, ^48^ ROS is a double‐edged sword and the influence of ROS within cancer cells, whether they are favourable or harmful, may depend on several factors, such as cell type, stimulus, duration, specificity and levels of ROS.

The overexpression of the oncogene HIF‐1 in cancer cells results in their adaptation to a microenvironment that is hypoxic compared to normal cells, thereby avoiding apoptosis. The mechanism of action of HIF‐1 includes a switch from oxidative phosphorylation to glycolysis, increasing glycogen synthesis and a switch from glucose to glutamine as the major substrate for fatty acid synthesis.^49^ It is worth noting that the phosphorylation of AKT results in the upregulation of HIF1 in cancer cells.^50^ Consistent with these results, we found that PD and 2‐DG co‐treatment increased HIF‐1α by inhibit the PI3K/AKT pathway in breast cancer cell lines (Figure 5). The glycolysis rate strongly depends on the upregulated expression and activity of glucose transporters (Gluts), such as Glut1, which is regulated by HIF‐1α and is considered the main overexpressed Glut, with a 10‐12‐fold higher expression in tumours than in normal cells.^49^, ^51^ In this study, the expression levels of Glut 1, LDH‐A and HK2 were detected to assess the glycolysis rate and the results indicated that PD and 2‐DG co‐treatment inhibited glycolysis in breast cancer cell lines. In addition, HK2 is the direct target of HIF‐1α in HCC cells.^52^ In this study, this is evidenced by the silencing of HK2 by siRNA enhancing the inhibitory effect of combination treatment on glycolysis and growth (Figure 5).

In line with our in vitro data, PD and 2‐DG co‐treatment potently suppressed tumour growth in murine syngeneic breast tumour models. Collectively, we first determined the synergistic anti‐cancer effects of PD and 2‐DG coutreatment and found that the effect was mediated by the ROS/PI3K/AKT/HIF1α/HK2 and glycolysis pathways in breast cancer. This study is the first to indicate the efficient combination treatment of PD and 2‐DG, providing a potential treatment strategy for breast cancer patients.

## CONFLICT OF INTEREST

The authors declare no competing financial interests.

## AUTHOR CONTRIBUTIONS

TZ, XZ and GD, CQ conceived and designed the experiments. TZ, HW, KJ and CQ performed the experiments. TZ, XZ, HW and CQ analyzed the data. TZ, GZ, GD and CQ wrote the paper. All authors read and approved the final manuscript.
